# Chemical Profiling of Nyaope and Its Public Health Implications

**DOI:** 10.3390/toxics14050410

**Published:** 2026-05-09

**Authors:** Lufuno Ratshisusu, Omphile E. Simani, Nakisani B. Moyo, Lufuno G. Mavhandu-Ramarumo, Ntakadzeni E. Madala, Jason T. Blackard, Selokela G. Selabe

**Affiliations:** 1 HIV and Hepatitis Research Unit, Department of Medical Virology, Sefako Makgatho Health Sciences University, Pretoria 0208, South Africa; omphile.simani@smu.ac.za (O.E.S.); lufuno.mavhandu-ramarumo@smu.ac.za (L.G.M.-R.); blackajt@ucmail.uc.edu (J.T.B.); gloria.selabe@smu.ac.za (S.G.S.); 2 Department of Food Science and Technology, Faculty of Science, Engineering and Agriculture, University of Venda, Thohoyandou 0950, South Africa; barbara.moyo@univen.ac.za; 3 Center of Excellence in Mass Spectrometry for Southern Africa, Faculty of Science, Engineering and Agriculture, University of Venda, Thoyondou 0950, South Africa; ntakadzeni.madala@univen.ac.za; 4 Division of Gastroenterology & Hepatology, College of Medicine, University of Cincinnati, Cincinnati, OH 45267-0595, USA; 5 Center for Addiction Research, University of Cincinnati, Cincinnati, OH 45267-0595, USA; 6 National Health Laboratory Service, Department of Medical Virology, Sefako Makgatho Health Sciences University, Pretoria 0208, South Africa

**Keywords:** nyaope, illicit drug analysis, UHPLC-qTOF-MS, molecular networking, computational metabolomics, mass spectrometry, South Africa

## Abstract

Nyaope is a highly addictive street drug that is widely used in South Africa, particularly in urban and peri-urban settings. Although it is traditionally consumed by smoking, increasing injection use has raised serious public health concerns due to an elevated risk of bloodborne viral infections and other drug-related health complications. The composition of nyaope is highly variable, frequently adulterated, and continually evolving, thus highlighting the need for detailed chemical characterization to support forensic investigations and public health interventions. An exploratory study design was conducted using eight nyaope samples seized from six sites within the City of Tshwane Metropolitan Municipality that were provided by the South African Police Service Forensic Science Chemistry Laboratory (SAPS-FSCL). Samples were analyzed using Ultra-High-Performance Liquid Chromatography coupled to Quadrupole-Time-of-Flight Mass Spectrometry (UHPLC-qTOF-MS) operated in data-dependent acquisition mode under positive ionization. Raw data from the methanolic extracts of nyaope was converted to mzML format and processed using SIRIUS software for compound annotation based on isotope pattern ranking and fragmentation analysis. Chemical profiling revealed multiple opiate-related compounds, including noscapine, heroin, papaverine, and codeine. Molecular networking revealed chemically diverse yet structurally related metabolites consistent with a poppy-derived botanical origin. In addition, multiple synthetic pharmaceutical adulterants were detected. Notably, one sample contained formaline, a toxic rodenticide structurally related to protopine, highlighting the risk of misidentification using less advanced analytical approaches. This study demonstrates the value of advanced computational metabolomics, including molecular networking and machine-learning-assisted mass spectrometry interpretation, for comprehensive characterization of complex illicit drug mixtures. These approaches enhance forensic accuracy and support informed public health and law-enforcement responses.

## 1. Introduction

Over the past two decades, there has been a rapid emergence of new psychoactive substances (NPS), with novel compounds consistently being introduced into the global illicit drug market and contributing to their increased use and availability worldwide [1,2,3,4,5]. This trend presents an ongoing public health concern [3,6]. One such substance is nyaope, a relatively new street drug that is believed to be a cocktail mixture containing low-grade heroin mixed with various adulterants, including milk powder, rat poison, bicarbonate of soda, cannabis, and, in some cases, antiretroviral drugs [7,8,9].

Nyaope is a highly addictive drug, with many users experiencing long-term dependence and facing significant health and social challenges as a result [10]. It is predominantly used in South Africa, especially in urban and peri-urban communities. The drug emerged between 2000 and 2006 in the Tshwane townships of Mamelodi, Soshanguve, and Atteridgeville [6]. Although it is typically smoked, injection use has become increasingly common in recent years. The composition of nyaope is highly variable, has evolved, and is largely influenced by the source and method of preparation [8,9]. It typically appears as a brown-coloured powder, often disguised as soil or cement, which complicates detection and law enforcement.

A study conducted in Pretoria, South Africa, identified nyaope samples containing both opiates and synthetic opioids [8]. Opiates are derived from the opium poppy plant (*Papaver* spp.), including compounds such as morphine, heroin, and codeine, whereas opioids are often synthetic or semi-synthetic analogues that share similar pharmacological effects [8]. In addition to these, non-opiate substances were detected in the same study, including central nervous system depressants (e.g., phenobarbitone, benzodiazepines) and stimulants (e.g., amphetamines, pipradol, and fenethylline) [8]. A mini review by Khine and Mokwena suggested that many of these substances share similar metabolic pathways, especially through cytochrome P450 enzymes, which can prolong drug effects and contribute to synergistic pharmacological responses [7]. These interactions likely intensify euphoria and contribute to the unique withdrawal symptoms reported in nyaope users.

Opioid use disorder is associated with substantial morbidity and mortality, with an estimated mortality risk that is 15 times higher than that of the general population [11,12]. In South Africa, this is compounded by the widespread use of nyaope which is linked to multiple adverse health outcomes, including an increased risk of blood-borne viral infections such as human immunodeficiency virus (HIV) and hepatitis C virus (HCV), largely due to unsafe injection practices and needle sharing [5,13,14,15,16,17]. In addition to overdose-related deaths, individuals who use nyaope often experience chronic health complications, social marginalization, and economic instability, all of which exacerbate their vulnerability [12,18]. Despite the growing public health concern, access to effective harm-reduction strategies, addiction treatment, and viral infection management remains limited in many South African communities.

Chronic use of nyaope has been associated with severe neurological damage. A South African study demonstrated fronto-temporal cortical atrophy among long-term users, implicating key brain regions responsible for cognition, emotion, and self-awareness [19]. This may explain the high burden of mental health issues observed in this population [20]. Collectively, these findings highlight the importance of continually monitoring the chemical makeup of nyaope to better understand its evolving risks and to inform public health responses [4]. Currently, there is no validated or standardized method for determining the composition of nyaope. Moreover, existing laboratory instruments capable of identifying its components are limited and often prohibitively expensive. Nevertheless, studies conducted in Gauteng, South Africa, have successfully identified its components using advanced analytical tools [8,21]. Given the complex, evolving nature of nyaope and the limitations of conventional forensic techniques, there is a critical need for advanced analytical techniques that provide a more detailed and comparative chemical profile. Therefore, this study applied the Ultra-High-Performance Liquid Chromatography coupled to Quadrupole-Time-of-Flight Mass Spectrometry (UHPLC-qTOF-MS) in combination with molecular networking and sum formula identification by ranking isotope patterns using mass spectrometry (SIRIUS) software to characterize the chemical composition of contemporary nyaope samples and to compare these results with earlier reports to assess the evolution of its composition over time, with implications for forensic toxicology and harm reduction strategies.

## 2. Materials and Methods

### 2.1. Sample Collection and Preparation

**Figure 1:**
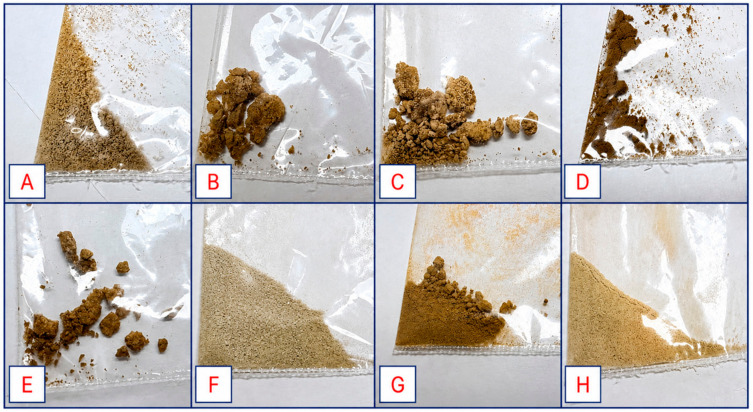
Nyaope samples collected from six areas within the City of Tshwane Metropolitan Municipality. (**A**) Soshanguve sample; (**B**) Mabopane sample; (**C**,**D**) Atteridgeville samples; (**E**) Pretoria sample; (**F**,**G**) Mamelodi samples; and (**H**) Ga-Rankuwa sample. The powder samples show varying colours, reflecting differences in adulterants and formulation.

This was an exploratory study design, constrained by the limited availability of nyaope samples (*n* = 8), and therefore intended to provide high-resolution chemical profiling rather than a comprehensive quantitative assessment of drug composition across regions. Compound annotation was performed using Global Natural Products Social Molecular Networking 2.0 (GNPS2) molecular networking and SIRIUS 6.1.0, an in silico tool. Identifications were assigned at confidence level 2 in accordance with Metabolomics Standards Initiative (MSI) guidelines. A total of eight heroin powder (nyaope) samples that were seized by the South African Police Service (SAPS) from six areas within the City of Tshwane Metropolitan Municipality between 2020 and 2024 were obtained from the SAPS Forensic Science Chemistry Laboratory (SAPS-FSCL). As shown in Figure 1, the samples originated from Soshanguve (Figure 1A; *n* = 1), Mabopane (Figure 1B; *n* = 1), Atteridgeville (Figure 1C,D; *n* = 2), Pretoria Central (Figure 1E; *n* = 1), Mamelodi (Figure 1F,G; *n* = 2), and Ga-Rankuwa (Figure 1H; *n* = 1).

For nyaope sample analysis, powdered samples (approximately 0.10 g each) were dissolved in 1.2 mL of 80% methanol (1:10 *m*/*v*). The mixtures were incubated overnight at 70 rpm using a digital tube rotator (DLAB Scientific, Beijing, China).

### 2.2. Chromatographic Separation and Mass Spectrometric Analysis

Following extraction, the solutions were filtered through 0.22 μm nylon syringe filters into amber ultra-high-performance liquid chromatography (UHPLC) vials for subsequent analysis using quadrupole time-of-flight mass spectrometry (qTOF-MS) (LC-MS-9030 model, Shimadzu Corporation, Kyoto, Japan) according to a modified method established by Moyo and colleagues [22]. Compounds were separated using a Kinetex C_18_ column (100 × 2.1 mm, 1.7 µm) (Phenomenex, Separations, Johannesburg, South Africa) maintained at 55 °C. A volume of 3 µL was injected into the instrument and the compounds in nyaope extracts were separated using a binary mobile phase gradient elution at a flow rate of 0.3 mL min^−1^. The mobile phases A and B contained 0.1% (*v*/*v*) formic acid in ultrahigh purity water and methanol, respectively. From 0–8 min, the mobile phase composition was 5%. The composition of mobile phase B was increased to 40% at 8 min and then to 95% between 23 and 25 min. At 27 min, the gradient was altered to 5% mobile phase B and was held at this composition until 30 min, for column re-equilibration. A quadrupole time-of-flight (qTOF) high resolution mass spectrometer with an electrospray ionization (ESI) interface operating in positive ionisation mode was employed for mass spectral analysis. The parameters of the mass spectrometer were set as follows: interface voltage of 4.0 kV, interface temperature of 300 °C, nebulization and dry gas flow of 3 L min^−1^, heat block temperature of 400 °C, DL temperature of 280 °C, detector voltage of 1.8 kV, and flight tube temperature of 42 °C. To monitor high mass accuracy, sodium iodide ion clusters were utilized as a calibrant. MS^1^ and MS^2^ were generated concurrently [using data-dependent acquisition (DDA)] for all ions with *m*/*z* values ranging from 100 to 1000 and an intensity threshold greater than 3000. Argon was used as a collision gas in the MS^2^ runs with a collision energy of 30 eV. LC-MS grade methanol and water were purchased from Romil SpS (Cambridge, UK).

### 2.3. Feature-Based Molecular Networking and Metabolite Annotation

A feature-based molecular network was constructed using the online workflow available on the GNPS2 website (http://gnps2.org). The raw data acquired from UHPLC-qTOF-MS, was converted first to mzML format before being uploaded to mass spectrometry–data independent analysis (MS-DIAL) for pre-processing. The data was uploaded to MS-DIAL version 4.7, the MS^1^ and MS^2^ tolerances were set to 0.02 Da, and the retention time tolerance was set to 0.05 min. The mgf and feature table files were exported to GNPS2 for feature-based molecular networking. The precursor ion mass tolerance and MS/MS fragment ion tolerance were both set to 0.02 Da. Matching minimum fragments, and the cosine score were set at 4 and 0.6, respectively. The resulting molecular network was visualized using the GNPS2 web browser, Cytoscape (v3.10.3), and SIRIUS (v6.1.0) was utilized to predict fragmentation patterns of the compounds of interest in this study [23]. Data in mgf format was imported to SIRIUS 6.1.0. For molecular formula identification, positive ionisation was selected for possible ionisation and the elements that were allowed in the molecular formula were C, H, N, P, and O. Structural database search was performed using GNPS2, Human Metabolome Database (HMDB), Knapsack, Coconut, Chemical Entities of Biological Interest (ChEBI), and Natural products. Canopus was utilized for compound class prediction.

## 3. Results

### 3.1. Chemical Profiling of Nyaope Samples

**Figure 2:**
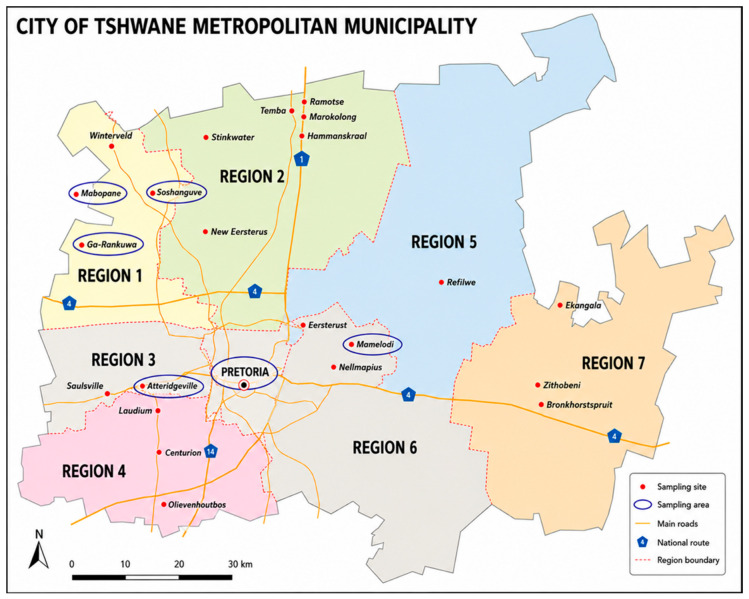
A map representation of the City of Tshwane Metropolitan Municipality. Circled are the areas where the nyaope samples were collected [24].

Eight nyaope samples were obtained through the SAPS-FSCL and originated from six locations within the City of Tshwane Metropolitan Municipality, including Soshanguve, Mabopane, Ga-Rankuwa, central Pretoria, Mamelodi, and Atteridgeville (Figure 2).

**Figure 3:**
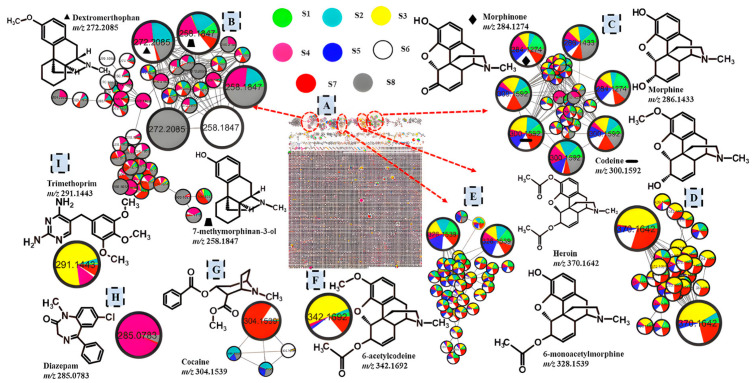
Infographic display of compounds identified in nyaope samples using UHPLC-qTOF-MS and feature-based molecular networking: (**A**) full network; (**B**–**F**) morphinan derivatives; (**G**) cocaine, a tropane alkaloid; (**H**,**I**) synthetic pharmaceutical drugs. Most of these compounds have previously been reported in *Papaver somniferum* (opium poppy) plants, suggesting that some nyaope constituents were derived from this plant. The different colours in the nodes represent nyaope samples collected from different areas. All the nodes of interest have been enlarged. Molecular families containing compounds of interest are circled on the full network and arrows are used to show their location on the figure.

In this study, the chemical composition of nyaope collected from six different areas in the City of Tshwane Metropolitan Municipality was profiled using the UHPLC-qTOF-MS and molecular networking. Notably, most of the identified compounds were detected across the majority of samples (Figure 3 and Figure S1), including multiple structurally related morphinan alkaloids (e.g., morphine, codeine, morphinone, heroin, 6-monoacetylmorphine, 6-acetylcodeine, oxycodone, and N-propargylnormorphine) and benzylisoquinoline alkaloids (e.g., noscapine, cotarnine, reticuline, papaverine, 3-O-acetyl-4′-O-demethylpapaveroxine, protopine, and narcotoline), as well as several unrelated psychoactive substances, pharmaceuticals, and adulterants (e.g., diazepam, trimethoprim, chloroquine, caffeine and acetaminophen). The distribution of different colours in each node in Figure 3 and Figure S1 provides a semi-quantitative overview based on the compound abundance across samples, highlighting regional differences. The identified compounds in different samples are shown in Table S1.

### 3.2. Identification of Morphinan Alkaloids

Among the identified molecules were 7-methylmorphinan-3-ol (*m*/*z* 258.1847) [M+H]^+^ and dextromethorphan (*m*/*z* 272.2085) [M+H]^+^ which both share the characteristic morphinan tetracyclic scaffold, explaining their clustering in the same molecular family within the molecular network (Figure 3B). Both these compounds showed fragment ions at *m*/*z* 171.0798 and 147.0797 which are indicative of loss of the dimethylamino group and part of the methoxy phenyl moiety and, O-demethylation after the opening of the C ring, respectively [25]. The nyaope samples also contained other illicit morphinan compounds including morphine, codeine, morphinone (Figure 3C), heroin (Figure 3D), and 6-monoacetylmorphine (Figure 3E) at *m*/*z* 286.1433, 300.1592, 284.1274, 370.1642, and 328.1539 [M+H]^+^, respectively. These compounds did not all cluster together using molecular networking, probably due to the different modifications on the morphinan core structure, such as the presence of hydroxyl, methoxy, ketyl and acetyl groups in these compounds. A fragment ion at *m*/*z* 268.1329 for heroin has been reported to result from the loss of ketene (H2C=C=O) and acetic acid groups from this compound [26]. The subsequent cleavage of the piperidine ring resulting in the loss of an amine group (CH_2_CHNHCH_3_) has been reported to give rise to a base peak at *m*/*z* 211.0744 for this compound [26]. The peak at *m*/*z* 211.744 was also observed in morphine, codeine and 6-monoacetylmorphine in addition to a common fragment ion at *m*/*z* 165.0692, which is representative of loss of part of the morphinan core structure [27]. The ion at *m*/*z* 165.0692 is commonly used in multiple reaction monitoring (MRM) methods for quantifying or confirming the presence of these compounds since it is a diagnostic ion for morphine-like molecules [28,29]. The peak observed in morphinone at *m*/*z* 227.0697 is suspected to be due to the loss of an amine group (CH_2_CHNHCH), a neutral loss also observed in morphine and codeine, and this fragmentation pattern corresponds to what was previously reported [30]. Additional morphinan derivatives were putatively annotated using SIRIUS and these include 6-acetylcodeine at *m*/*z* 342.169 (Figure 3F) and oxycodone at *m*/*z* 316.1540 (Figure S1B), highlighting the benefits of using computational metabolomics tools for annotating compounds in complex mixtures of recreational drugs that can otherwise be easily missed using targeted approaches. These two compounds are semi-synthetic opioids reported to be produced during the illicit production of heroin from morphine [31]. Oxycodone showed a fragment ion at *m*/*z* 199.0752, which was reported to be a diagnostic peak for morphinans that have a tetrahydrofuran ring in their structure [25]. Although oxycodone is a pharmaceutical drug, it is highly addictive and often abused. A compound at *m*/*z* 310.1431 also tentatively identified using SIRIUS as N-propargylnormorphine (Figure S1I), which is also a morphine derivative.

### 3.3. Benzylisoquinoline Alkaloids

Several benzylisoquinoline alkaloids including noscapine (*m*/*z* 414.1542), cotarnine (*m*/*z* 220.0962), reticuline (*m*/*z* 330.1694), papaverine (*m*/*z* 340.1539), 3-O-acetyl-4′-O-demethylpapaveroxine (*m*/*z* 444.1651), protopine (*m*/*z* 354.1332) and narcotoline (*m*/*z* 400.1390) found in the opium poppy plant were also identified across different samples as shown in Figure S1C–H. Reticuline showed a major peak at *m*/*z* 192.1013 which was suspected to be due to the presence of a positively charged isoquinoline moiety, and the presence of this moiety with additional methylation was observed at *m*/*z* 202.0857 in papaverine [32]. Papaverine also showed a fragment ion at *m*/*z* 325.1307 which corresponds to the rearrangement of the C3′ and C4′ methoxy groups to a methylenedioxy bridge. Noscapine and narcotoline both showed a fragment ion at *m*/*z* 220.0963, which corresponds to an isoquinolinium-type cation formed after bond cleavage between the isoquinoline and the phthalide ring. Cortanine and noscapine shared a common fragment ion at *m*/*z* 205.0729 which could be due to the loss of a methyl group from the isoquinoline moiety [33]. It is notable that 3-O-acetyl-4′-O-demethylpapaveroxine at *m*/*z* 444.1651 also exhibited these two fragment ions (*m*/*z* 220.0630 and 205.0729) because it is structurally similar to noscapine. The base peak at *m*/*z* 188.0461 for protopine has been reported to be resulting from structural cleavage and subsequent loss of water from the isoquinoline fragment [34]. Cocaine, a tropane alkaloid was also identified in almost all the samples at *m*/*z* 304.1539 (Figure 3G) through its major fragment ions at *m*/*z* 182.1176 and 105.0329 which correspond to the neutral loss of benzoic acid and the presence of a benzoyl cation, respectively [35].

### 3.4. Synthetic Pharmaceutical Drugs

Notably, synthetic pharmaceutical drugs, diazepam (Figure 3G), trimethoprim (Figure 3H) and chloroquine (Figure S1K) were also found in the nyaope samples at *m*/*z* 285.0783, 291.1443 and 320.1884 [M+H]^+^, respectively. Diazepam was characterized by fragment ions at *m*/*z* 257.0834, 228.0581 and 193.088, which correspond to the sequential loss of CO, CH_2_=NH and CI, respectively [36,37]. The loss of formaldehyde (30 Da) from trimethoprim resulted in the fragment ion at *m*/*z* 261.0995, whereas the peak at *m*/*z* 123.0652 is representative of the methylenediaminopyrimidine moiety [38]. Methaqualone (*m*/*z* 251.117) a synthetic sedative–hypnotic drug was characterized by its fragment ion at *m*/*z* 132.0800 and 117.0568 which are suspected to be derived from the quinazolinone moiety of this compound [39]. Caffeine (*m*/*z* 195.0904) and acetaminophen (*m*/*z* 152.070) were also identified in all the eight samples as shown in Figure S1K,L and these have been reported to be used as cutting agents in illicit drug formulations [40]. Caffeine fragmented to give ions at *m*/*z* 138.0656 and *m*/*z* 110.0707, indicative of the loss of H_3_C-N=C=O and the subsequent loss of CO, respectively [41]. Acetaminophen displayed its typical fragmentation, yielding *m*/*z* 110.0594 from α-cleavage of the amide bond and partial loss of the acetyl group [42].

## 4. Discussion

This study provides one of the most detailed chemical characterizations of nyaope to date and is the first to apply UHPLC-qTOF-MS in combination with feature-based molecular networking and in silico fragmentation tools to profile nyaope samples from Pretoria, South Africa. The findings reveal that nyaope remains a chemically diverse and heterogeneous mixture, with most samples containing multiple morphinan alkaloids, benzylisoquinoline derivatives, and additional psychoactive or pharmaceutical agents. The consistent presence of morphine, codeine, heroin, 6-monoacetylmorphine, and related opium-poppy-derived constituents across samples suggests a common botanical origin and highlights the ongoing circulation of opioid-based formulations in urban and peri-urban communities. These results align with earlier work but also reflect the evolving composition of nyaope as new substances are introduced into the illicit drug market [8,12,21,43,44].

Importantly, these compounds share metabolic pathways and may interact synergistically through cytochrome P450 systems, potentially increasing toxicity, prolonging pharmacological effects, and complicating withdrawal [7]. Many constituents share metabolic pathways, particularly through cytochrome P450 enzymes, which increases the likelihood of unpredictable drug–drug interactions when poly-adulteration occurs. Additives such as diazepam may mask early signs of opioid toxicity by reducing agitation or seizures, yet they simultaneously exacerbate respiratory depression, thereby increasing lethality [45,46]. Similarly, chloroquine introduces cardiotoxic effects, including QT prolongation and arrhythmias that overlap with opioid-induced hypoxia and circulatory collapse, complicating clinical recognition and management [47]. Therefore, these overlapping metabolic and clinical effects highlight the urgent need for advanced detection workflows and integrated harm-reduction strategies [48,49].

For people who use nyaope, these chemical interactions may exacerbate overdose risk, deepen physiological dependence, and intensify relapse episodes. Moreover, molecular networking was applied for the first time to these samples, demonstrating that they contain chemically diverse and complex metabolites. The application of molecular networking revealed clusters of structurally related compounds, offering insights into biosynthetic origins and processing pathways that would be difficult to detect using targeted analytical methods. Several semi-synthetic opioids including oxycodone, 6-acetylcodeine, and N-propargylnormorphine which were identified only through computational dereplication, emphasizing the importance of advanced metabolomics tools for detecting emerging or low-abundance constituents. These compounds may act synergistically through overlapping metabolic pathways, potentially amplifying central nervous system depression, increasing toxicity, and contributing to the high dependency potential observed among nyaope users. Synthetic pharmaceuticals were detected; however, the dominant constituents were opium poppy-derived alkaloids.

Beyond opioid constituents, the presence of benzylisoquinoline alkaloids such as noscapine, papaverine, reticuline, protopine, and narcotoline further supports the use of opium-derived plant materials. The presence of protopine is consistent with the botanical origin of the heroin base material. Protopine is a naturally occurring benzylisoquinoline alkaloid found in Papaveraceae species, including *Papaver somniferum*. This reinforces the chemical complexity of street-level heroin formulations and highlights the variable composition experienced by users. These compounds were consistently detected across samples and clustered into distinct molecular families, as expected from their shared biosynthetic lineage. Notably, protopine exhibited structural similarity to formaline, the toxic rodenticide found in one sample, highlighting how less advanced analytical approaches might misidentify hazardous adulterants. Even in a single sample, the presence of formaline, is a major public health concern given its severe toxicological profile. The detection of non-opioid pharmaceuticals including diazepam, chloroquine, trimethoprim, methaqualone, caffeine, and acetaminophen illustrates the extent of chemical adulteration in nyaope formulations. Their presence is concerning as they can mask overdose symptoms, contribute to polypharmacy toxicity, complicate emergency management of opioid-induced emergencies, or produce unpredictable pharmacodynamic interactions. While some additives (e.g., caffeine, acetaminophen) are commonly used as bulking agents in illicit drugs, others such as chloroquine or trimethoprim have no known psychoactive value and may either reflect contamination, attempts to mask withdrawal symptoms, or intentional adulteration to increase volume and profitability [50,51]. The findings highlight the urgent need for strengthened surveillance systems capable of rapidly detecting emerging adulterants and toxic compounds in street drugs that are circulating within vulnerable communities.

The detection of cocaine (stimulant alkaloid), diazepam (benzodiazepine), trimethoprim (antibiotic), chloroquine (antimalarial), and methaqualone (sedative-hypnotic), in the nyaope samples suggests the presence of multiple non-opioid adulterants within the formulations. Acetaminophen (paracetamol) and caffeine are commonly used to dilute or “bulk up” street drugs. These substances may be intentionally added by dealers to increase the bulk of the heroin (nyaope) powder, enhance or modify its psychoactive effects, or create the perception of heightened potency, thereby improving the product’s marketability. In addition to intentional adulteration, some compounds may also reflect contamination that is introduced at various stages of the illicit manufacturing or handling process. Collectively, the variability and pharmacological diversity of these adulterants underscore the unpredictable toxicity associated with nyaope use and highlight significant public health concerns, particularly for users who may be unaware of the composition of the substances that they consume.

The chemical diversity observed in this study has important implications for both harm-reduction initiatives and clinical care. Given the unpredictable composition of nyaope, individuals who smoke or inject the drug face compounded health risks including overdose, respiratory depression, cardiovascular complications, and exposure to toxic adulterants. Prior studies have already linked nyaope use to severe clinical outcomes such as respiratory distress, neonatal complications, and infective endocarditis among individuals injecting the drug. The present findings reinforce concerns that adulteration practices may exacerbate these risks and contribute to the high morbidity observed among users. From a public health perspective, the chemical heterogeneity observed in these nyaope samples intersects with multiple layers of risk. People who use nyaope—particularly those who inject it—face heightened vulnerability to HIV, HCV and HBV, overdose, mental health complications, and social marginalization [1,3,13,16,52,53]. The presence of strong opioids and toxic adulterants increases the likelihood of both fatal and non-fatal overdoses, while the variability of composition across regions complicates clinical management and harm-reduction interventions. Furthermore, the evolving formulation of nyaope underscores the need for early-warning systems and real-time drug-checking services, which could support safer-use practices, improve linkage to care, and empower communities disproportionately affected by substance use.

South Africa currently lacks standardized forensic or public health frameworks for routinely monitoring the chemical composition of nyaope or other emerging drug mixtures. As shown in this study, conventional analytical approaches are insufficient to detect the full spectrum of psychoactive compounds and adulterants. Integrating metabolomics-based approaches into forensic laboratories and harm-reduction settings could provide a more comprehensive understanding of the evolving drug market, support evidence-based interventions, and guide resource allocation for addiction treatment services, overdose prevention, and community education. Additionally, strengthening collaboration between forensic science units, substance-use treatment programs, and public health authorities is essential for developing coordinated responses that address both chemical risks and the social drivers of drug use. Therefore, drug abuse is considered a chronic disease because individuals continue to seek and use drugs uncontrollably, despite harmful consequences. It is classified as a brain-related disorder, as repeated drug use alters brain structure and function, impairing self-control and creating an intense, persistent craving for the substance. Addiction can also lead to harmful behaviours, including theft, violence, and other social consequences. Furthermore, even after prolonged recovery, individuals remain at considerable risk of relapse. These features are particularly evident in nyaope abuse, highlighting its role as a relapsing, chronic disease that is difficult and costly [1,19,54,55,56,57,58]. Substance abuse, including alcohol and prescription drugs, can elicit symptoms resembling mental illness during both intoxication and withdrawal. In cases of amphetamine or cocaine abuse, depression or persistent psychosis may continue long after detoxification, contributing to chronic psychiatric disorders [59]. According to previous reports, cocaine addiction poses serious health risks and can be fatal due to unpredictable cardiotoxic effects [60]. In addition, chronic nyaope use is associated with cardiovascular complications, including hypertension, cardiomyopathy, myocarditis, arrhythmias, and myocardial infarction, as well as cerebrovascular aneurysms [60].

Nyaope is a complex street drug commonly containing a mixture of opioids and other psychoactive substances. Among other opioid components, oxycodone, morphine, methadone, fentanyl, and buprenorphine are classified as strong opioids and are primarily used in clinical settings to manage severe pain [60]. In contrast, weaker opioids such as tramadol, propoxyphene, and codeine are generally prescribed for moderate to mild pain relief [60]. However, misuse of these opioids can lead to severe toxicity, with respiratory depression and over-sedation being hallmark features of fatal opioid poisoning. Beyond their pharmacological effects, nyaope and its opioid constituents have been associated with a range of serious health complications. Cardiovascular effects include arrhythmias, myocarditis, and cardiomyopathy, with a case report describing three instances of tricuspid valve infective endocarditis associated with intravenous nyaope use [15,56]. Respiratory complications have also been reported, including upper and lower airway obstruction and recurrent pneumonias among infants exposed to nyaope prenatally [61]. Moreover, another case report highlighted multiple neonatal complications in infants born to nyaope-using mothers, including retinopathy of prematurity, jitteriness, generalized tonic–clonic seizures, symmetric growth restriction, excessive sucking movements, and hypoglycemia [62]. Collectively, these findings underscore the severe systemic and developmental consequences associated with nyaope abuse.

Overall, findings from this study highlight that nyaope remains a dynamic and unpredictable drug mixture with substantial health risks. The incorporation of advanced computational metabolomic tools demonstrates significant value for characterizing this complexity and identifying compounds that may otherwise be overlooked. Continued monitoring of nyaope composition is essential to inform harm-reduction strategies, clinical management, and public health policy, particularly as new psychoactive substances emerge, and formulations continue to evolve within the illicit drug market. Most of the compounds identified in nyaope were alkaloids and morphinans that have been reported in opium poppy plants indicating that parts of these formulations were prepared from constituents of this plant. Also, a lot of compounds remain unknown in these formulations, which is a general challenge in metabolomics studies as many compounds remain as dark matter. This challenge is further exacerbated by the complexity of most recreational formulations and their evolving nature [63], as there is no standard formulation, particularly for street recreational drugs. Therefore, findings of this study highlight the complexity of nyaope formulations that require advanced computational approaches such as molecular networking and a fragmentation predicting tool (SIRIUS) to uncover their chemical composition. These approaches enable rapid dereplication of known compounds and discovery of novel constituents as formulations evolve over time and across sources. By visually mapping the entire chemical space of a given sample and propagating annotations to related unknowns, molecular networking can be a powerful strategy in accelerating forensic monitoring and investigations of illicit drugs.

The structural similarity between protopine and formaline presents a notable analytical challenge, particularly when using lower-resolution techniques such as conventional Gas Chromatography–Mass Spectrometry (GC-MS), which rely heavily on nominal mass and limited fragmentation patterns, which may not adequately resolve compounds with overlapping fragment ions or similar molecular masses as previously described [64,65]. This limitation can increase the likelihood of misidentification, particularly in complex matrices such as nyaope where co-elution and compound heterogeneity are common. In contrast, high-resolution mass spectrometry such as UHPLC-qTOF-MS and computational metabolomics tools including, feature-based molecular networking and SIRIUS improve compound annotation. High-resolution mass spectrometry enables accurate mass determination and improved fragmentation profiling, while molecular networking facilitates clustering of structurally related compounds based on spectral similarity. In addition, in silico tools enhance annotation confidence through molecular formula and fragmentation pattern prediction [66,67]. Collectively, the results highlight the limitations of conventionally targeted toxicology testing, which would likely fail to detect many of these structurally diverse components. The use of molecular networking and computational annotation tools (e.g., SIRIUS) proved critical for resolving complex mixtures, identifying novel or uncommon derivatives, and understanding chemical relationships among detected compounds. This analytical depth is essential for informing public health surveillance, addiction treatment, and forensic investigations in regions with evolving drug formulations such as nyaope.

## 5. Recommendations

Based on the findings of this study, we propose several recommendations. First, future investigations should incorporate quantitative analyses of identified compounds to determine concentration ranges and more accurately assess potential toxicity. Second, larger and more geographically diverse sampling efforts are needed to capture the full variability and evolving composition of nyaope across South Africa. Third, forensic and public health laboratories should consider the routine integration of advanced computational metabolomic tools, such as molecular networking and SIRIUS, to enhance monitoring and early detection of new psychoactive substances and adulterants. Finally, enhanced collaboration between forensic scientists, public health agencies, and harm-reduction programmes is crucial to ensure that evolving nyaope formulations inform targeted prevention, treatment, and policy interventions.

## 6. Limitations

This study has several limitations. First, the relatively small sample size limits the generalizability of the findings and may not fully capture the variability in nyaope composition across different settings. Second, the study employed an untargeted, qualitative metabolomics approach and did not include quantitative measurements of compound concentrations, which restricts the ability to assess toxicological relevance. Third, compound identification was based on putative annotation using molecular networking and in silico tools (SIRIUS), without confirmatory validation using reference standards. As such, identifications correspond to MSI level 2 (putatively annotated compounds) and should be interpreted with caution. Future studies incorporating larger sample sizes, targeted quantification, and confirmatory analyses are warranted.

## 7. Conclusions

This study provides a comprehensive chemical profile of nyaope using UHPLC-qTOF-MS and molecular networking, revealing a highly heterogeneous mixture dominated by morphinan and benzylisoquinoline alkaloids alongside unexpected pharmaceutical adulterants. The presence of potent opioids, toxic additives, and structurally related derivatives underscores the health risks associated with nyaope use and its unpredictable composition. These findings highlight the need for improved drug-checking systems and surveillance tools to monitor emerging substances in South Africa’s illicit drug markets. Strengthening harm-reduction strategies and public health responses is essential to mitigate the severe social and health consequences linked to nyaope use.

## Data Availability

The data supporting the findings of this study are available from the corresponding authors upon reasonable request.

## Supplementary Materials

The following supporting information can be downloaded at: https://www.mdpi.com/article/10.3390/toxics14050410/s1, Table S1: Putative identification of compounds in nyaope using the UHPLC-qTOF-MS and feature-based molecular networking; Figure S1: Infographic display of additional compounds identified in nyaope through the UHPLC-qTOF-MS and feature-based molecular networking with (A) showing the full network, (B) oxycodone, (C–I) benzylisoquinoline alkaloids, (J) morphine derivative, (K,L) synthetic pharmaceutical drugs and (M,N) compounds that have been reported as cutting agents in illicit drug formulations.

## Abbreviations

The following abbreviations are used in this manuscript:

UHPLC-qTOF-MS	Ultra-high-performance liquid chromatography coupled with quadrupole time-of-flight mass spectrometry
UHPLC	Ultra-high-performance liquid chromatography
qTOF-MS	quadrupole time-of-flight mass spectrometry
qTOF	quadrupole time-of-flight
GC-MS	Gas Chromatography–Mass Spectrometry
MS	Mass Spectrometry
SIRIUS	Sum formula Identification by ranking isotope patterns using mass spectrometry
GNPS2	Global Natural Products Social Molecular Networking 2.0
ESI	Electrospray ionization
DL temperature	Desolvation Line temperature
DDA	Data-dependent Acquisition
mzML	mass spectrometry Markup Language
MS-DIAL	Mass Spectrometry–Data Independent Analysis
MRM	Multiple Reaction Monitoring
HMDB	Human Metabolome Database
ChEBI	Chemical Entities of Biological Interest
*m*/*z*	mass-to-charge ratio
mgf	mascot generic format
rpm	revolutions per minute
mL	millilitres
μm	micrometres
mm	millimetres
g	gram
min	minute
(*v*/*v*)	volume/volume
eV	electron volt
kV	kilovolts
°C	degree Celsius
L min	Litres per minute
NPS	New Psychoactive Substances
HIV	Human Immunodeficiency Virus
HCV	Hepatitis C Virus
SAPS	South African Police Service
SAPS-FSCL	South African Police Service-Forensic Science Chemistry Laboratory
SAHPRA	South African Health Products Regulatory Authority
SMUREC	Sefako Makgatho Health Sciences University Research Ethics Committee
PRF	Poliomyelitis Research Foundation
NRF	National Research Foundation
