# Supplementary material for: Chemical Profiling of Nyaope and Its Public Health Implications
Source: Toxics. 2026 May 9;14(5):410. doi: 10.3390/toxics14050410 (PMC13211222; doi:10.3390/toxics14050410)
Supplement: Supplementary file 1 [file toxics-14-00410-s001.zip › toxics-4288511-supplementary.pdf]

Supplementary Materials

Table S1. Putative identification of compounds in nyaope using the UHPLC-qTOF-MS and feature based molecular networking.

Sample ID	Compound name	tR (min)	Molecular ion mass	Molecular ion formula	Fragment ions	S1 (Mabopane)	S2 (Pretoria Central)	S3 (Mamelodi)	S4 (Mamelodi East)	S5 (Soshanguve)	S6 (Ga-Rankuwa)	S7 (Atteridgeville-1)	S8 (Atteridgeville-2)
**1**	Noscapine	11.05	414.1542	C22H23NO7	353, 323, 220, 205	✓	✓	✓	✓	✓	✓	✓	✓
**2**	6-Monoacetylmor-phine	7.92	328.1539	C19H21NO4	268, 211, 193, 183, 165	✓	✓	✓	✓	✓	✓	✓	✓
**3**	Dextromethorphan	9.20	272.2085	C18H25NO	215, 213, 171, 150, 147	✓	✓	✓	✓	✓	✓	✓	✓
**4**	*Chloroquine	5.47	320.1884	C15H29CIN3	205, 191, 179, 142	✓	-	✓	✓	-	✓	✓	✓
**5**	Diacetylmorphine (Heroin)	12.32	370.1642	C21H23NO5	328, 268, 211	✓	✓	✓	✓	✓	✓	✓	✓
**6**	Cotarnine	8.55	220.0962	C12H13NO3	205, 147	✓	✓	✓	✓	✓	✓	✓	✓
**7**	Morphine	3.04	286.1433	C17H19NO3	237, 229, 211, 201, 185, 165, 155	✓	✓	✓	✓	✓	✓	✓	✓
**8**	*Caffeine	7.07	195.0904	C8H10N4O2	138, 123, 110	✓	✓	✓	✓	✓	✓	✓	✓
**9**	Codeine (methylmor-phine)	4.72	300.1592	C18H21NO3	237, 209, 194, 121, 107	✓	✓	✓	✓	✓	✓	✓	✓

**10**	Protopine	8.25	354.1332	C20H19NO5	188, 173	✓	✓	✓	✓	✓	✓	✓	✓
**11**	Reticuline	7.16	330.1694	C19H23NO4	192, 177, 143, 137	✓	-	✓	✓	✓	✓	✓	✓
**12**	*Cocaine	7.48	304.1539	C17H21NO4	229, 199, 185, 183, 181, 175, 173, 153	✓	✓	✓	-	✓	✓	✓	✓
**13**	Papaverine	8.80	340.1539	C20H21NO4	324, 296, 202, 171	✓	✓	✓	✓	✓	✓	✓	✓
**14**	7-Methylmorphinan-3-ol	7.66	258.1847	C17H23NO	213, 201, 199, 159, 171, 147, 133	✓	✓	-	-	✓	-	-	✓
**15**	*Trimethoprim	6.57	291.1443	C14H18N4O3	261, 230, 201, 123	✓	✓	✓	✓	✓	✓	-	✓
**16**	*Diazepam	14.40	285.0783	C16H13ClN2O	257, 241, 228, 193, 154	-	-	-	✓	✓	✓	✓	✓
**17**	*Acetominophen	0.96	152.0701	C8H9NO2	110, 109	✓	✓	✓	✓	✓	✓	✓	✓
**18**	6-hydroxy-4-meth-oxy-6-7,8-dihdro-di-oxoloisoquinolinyl-methy-2,3-dimetho-thybenzoic acid	6.83	432.165	C22H25NO8	381, 220, 205,	✓	✓	✓	✓	✓	✓	✓	✓
**19**	N-propargylnormor-phine	5.60	310.1431	C19H19NO3	253, 211,161	✓	✓	✓	-	✓	✓	✓	-

**20**	3-O-acetyl-4′-O-demethylpapaveroxine	7.71	444.1651	C23H25NO8	384, 220, 205	-	✓	-	✓	✓	-	-	✓
**21**	6-acetylcodeine	5.35	342.1692	C20H23NO4	243, 226, 197, 183, 165	✓	✓	✓	✓	✓	✓	✓	-
**22**	10-methoxy-4-methyl-12-oxa-4-azapentycyclooctadecapentaene	7.75	282.1484	C18H19NO2	266, 260, 225, 191, 175, 165	✓	✓	✓	✓	✓	✓	✓	✓
**23**	6,7,9-trimethoxy-3-methyl-hexahydro-methanobenzofuroisoquinolinone	6.64	358.1641	C20H23NO5	298, 241, 213, 181,	✓	✓	✓	✓	✓	✓	✓	✓
**24**	Narcotoline	10.20	400.1392	C21H21NO7	366, 339, 309, 220	-	-	✓	-	-	✓	✓	✓
**25**	Morphinone	2.51	284.1274	C17H17NO3	227, 225, 209, 197,181, 169	✓	✓	✓	✓	✓	✓	✓	✓
**26**	*Methaqualone	12.49	251.1173	C16H14N2O	132, 117	✓	✓	✓	✓	✓	✓	✓	✓
**27**	Oxycodone	3.15	316.154	C18H21NO4	199, 185, 177	✓	✓	✓	✓	✓	✓	✓	✓

Notes: S- Sample; tR = retention time; min = minutes; (-) = absence of the compound; (✓) = presence of the compound; Compounds annotated with an asterisk (*) indicate adulterants detected in the nyaope samples that do not originate from Papaver spp. (the opium poppy plant).

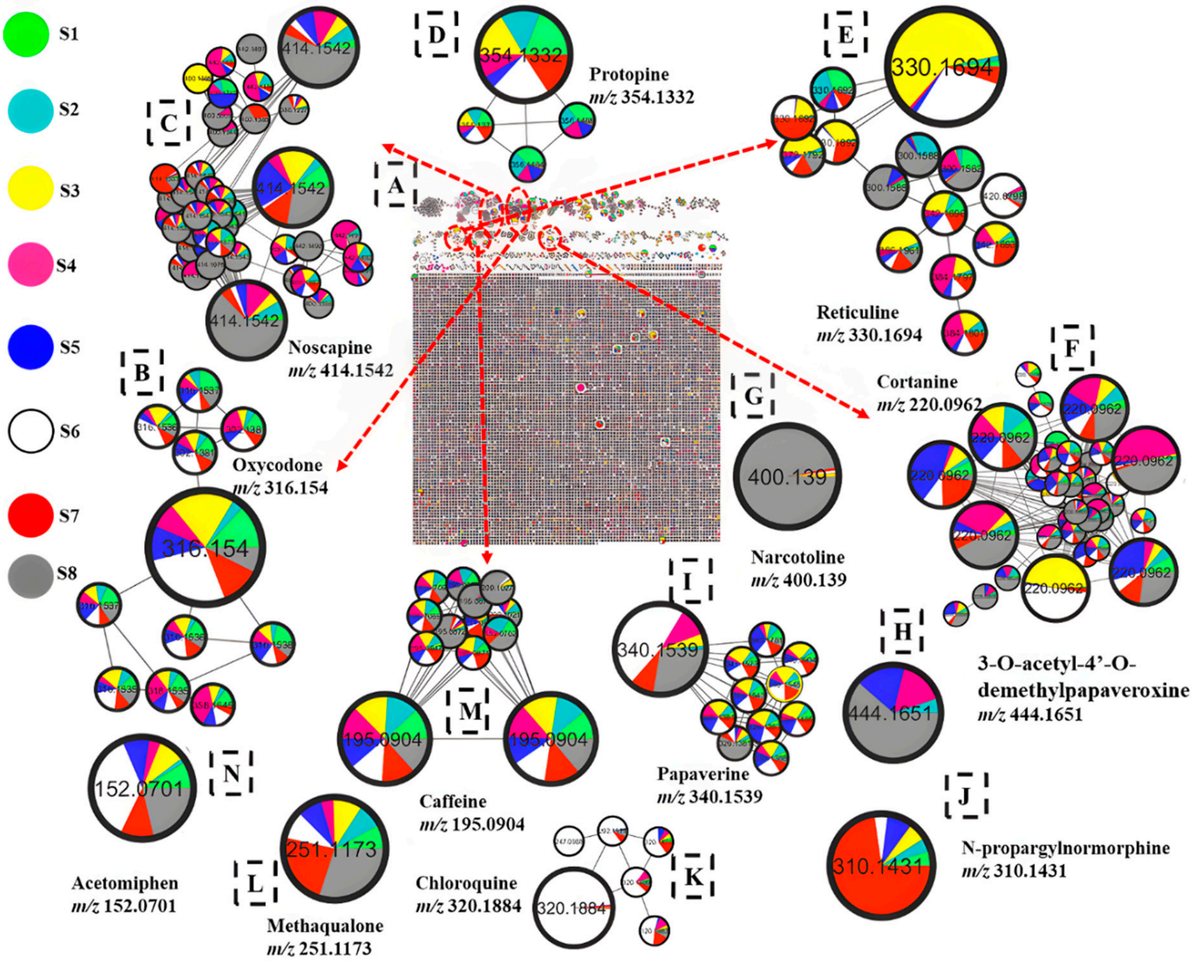

Figure S1. Infographic display of additional compounds identified in nyaope through the UHPLC-qTOF-MS and feature-based molecular networking with (A) showing the full network, (B) oxycodone, (C-I) benzylisoquinoline alkaloids, (J) morphine derivative, (K and L) synthetic pharmaceutical drugs and (M and N) compounds that have been reported as cutting agents in illicit drug formulations.
